# *PtychoShelves*, a versatile high-level framework for high-performance analysis of ptychographic data^1^

**DOI:** 10.1107/S1600576720001776

**Published:** 2020-03-13

**Authors:** Klaus Wakonig, Hans-Christian Stadler, Michal Odstrčil, Esther H. R. Tsai, Ana Diaz, Mirko Holler, Ivan Usov, Jörg Raabe, Andreas Menzel, Manuel Guizar-Sicairos

**Affiliations:** a Paul Scherrer Institute, 5232 Villigen PSI, Switzerland; b ETH and University of Zürich, Institute for Biomedical Engineering, 8093 Zürich, Switzerland

**Keywords:** ptychography, *PtychoShelves*, MATLAB, high-performance computing, phase retrieval, synchrotrons

## Abstract

Over the past decade, ptychography has been proven to be a robust tool for non-destructive high-resolution quantitative electron, X-ray and optical microscopy. It allows for quantitative reconstruction of the specimen’s transmissivity, as well as recovery of the illuminating wavefront. Additionally, various algorithms have been developed to account for systematic errors and improved convergence. With fast ptychographic microscopes and more advanced algorithms, both the complexity of the reconstruction task and the data volume increase significantly. *PtychoShelves* is a software package which combines high-level modularity for easy and fast changes to the data-processing pipeline, and high-performance computing on CPUs and GPUs.

## Abstract 2

A new computer program for analysing ptychographic data combines both high-level simplicity and high-performance computing on large-scale computing clusters. It is available with a royalty-free non-exclusive licence for academic and non-commercial purposes.

## 1. Introduction   

The achievable resolution of a standard microscope is commonly limited by the numerical aperture of the objective lens. This limitation is particularly severe in the hard X-ray regime, where the higher photon energies and thus shorter wavelengths would otherwise allow for a significantly increased resolution. Consequently, lens-less imaging systems have been of great scientific interest. Removing the objective, however, often comes at the cost of computational inefficiency, as lens-less imaging methods commonly rely on iterative reconstruction algorithms. One example is ptychography, a scanning coherent diffraction imaging technique. Its core ideas were introduced as early as 50 years ago (Hoppe, 1969 ▸) but in the past decade, with the incorporation of iterative phase-retrieval algorithms (Faulkner & Rodenburg, 2004 ▸), it has evolved into a well established method for achieving a quantitative and high-resolution representation of the sample’s transmissivity (Pfeiffer, 2018 ▸).

**Figure 1:**
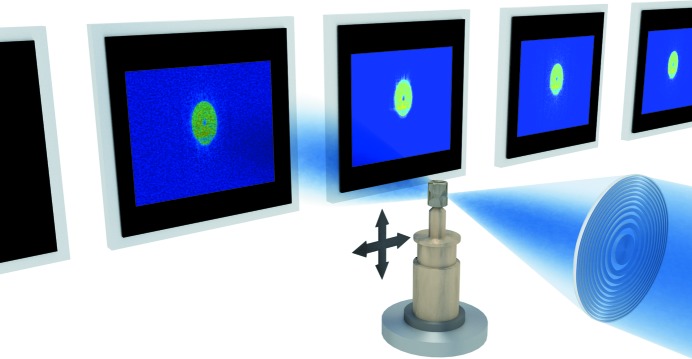
Conventional ptychography. A sample is scanned through coherent illumination such that the illuminated region partially overlaps with a previous data acquisition. For each sample position, a diffraction pattern is collected in the far-field regime.

In conventional ptychography, a diffracted intensity pattern is measured for each shift in the sample position with respect to the spatially and temporally coherent illumination, usually denoted as the probe (Fig. 1 ▸). The acquisition is done such that adjacent illumination regions partially overlap (Faulkner & Rodenburg, 2004 ▸; Edo *et al.*, 2013 ▸; da Silva & Menzel, 2015 ▸).

The wavefront directly after the sample, called the exit wave or view, Ψ_*j*_(**r**), at the *j*th position can be decomposed into



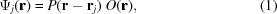
with **r** being the transverse real-space coordinate vector, *P*(**r** − **r**_*j*_) the illuminating probe and *O*(**r**) the sample transmissivity. The decomposition can be used to reconstruct both probe and sample transmissivity simultaneously (Rodenburg & Bates, 1992 ▸; Thibault *et al.*, 2008 ▸). The measured intensity *I*_*j*_(**q**) at the detector plane and the reciprocal-space coordinates **q** can be written as the propagated exit wave,
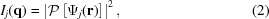
where 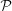 is the propagation operator. In conventional ptychography, also known as far-field ptychography because the diffracted intensity is measured in the far-field regime, the propagation operator can be reduced to a Fourier transform.

These ptychographic principles have been successfully applied to near-field geometries, also known as Fresnel ptychography (Stockmar *et al.*, 2013 ▸), as well as lens-based electron microscopes (Nellist & Rodenburg, 1998 ▸) and visible-light microscopes (Rodenburg *et al.*, 2007 ▸). In the optical regime, a variation of ptychography, termed Fourier ptychography, is nowadays more common (Zheng *et al.*, 2013 ▸), where the real-space shifts of the sample are substituted by displace­ments in reciprocal space of the object’s spectrum. Recently, Fourier ptychography has been demonstrated in the X-ray regime (Wakonig *et al.*, 2019 ▸). However, for the following discussion, the focus will be on conventional ptychography only, although the same principles can be generalized for other variations of ptychography methods by adjusting the propagation operator.

By recovering the complex exit wave for each scan position, not only the sample transmissivity but also the illuminating function can be reconstructed. In particular, the latter has proved to be crucial for ptychography to work at synchrotrons, as the illumination can deviate considerably from calculations (Thibault *et al.*, 2008 ▸). Moreover, the reconstructed probe can provide useful information about beam instabilities and partial coherence (Thibault & Menzel, 2013 ▸; Schropp *et al.*, 2010 ▸; Odstrčil *et al.*, 2016 ▸). In ptychography, the achievable resolution depends on the captured diffraction angle. Consequently, the detector readout, that is the number of pixels per diffraction pattern, is commonly chosen such that the effective pixel size at the sample plane is smaller than the expected resolution. The effective pixel size is given by
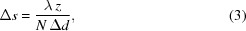
where λ denotes the illumination wavelength, *z* the distance from the sample to the detector, *N* the readout size of the detector frame and Δ*d* the detector pixel size.

For each ptychographic image, a scan is performed. At the cSAXS beamline at the Swiss Light Source (Paul Scherrer Institute, Switzerland), a ptychographic scan typically comprises between 10^2^ and 10^4^ scan positions and, depending on the energy, propagation distance, detector pixel size and achievable scattering angles, up to 1600 × 1600 pixels per diffraction pattern.

Various methods to recover the amplitude and phase of the sample and probe have been presented and tested over recent years (Faulkner & Rodenburg, 2004 ▸; Thibault *et al.*, 2008 ▸; Guizar-Sicairos & Fienup, 2008 ▸; Maiden & Rodenburg, 2009 ▸; Thibault & Guizar-Sicairos, 2012 ▸; Maiden *et al.*, 2017 ▸; Odstrčil *et al.*, 2018 ▸; Qian *et al.*, 2014 ▸). Despite the advances which have been achieved over the past decade, ptychography remains in continuous development and thus requires a flexible and modular computational framework to allow new algorithms and data-processing tools to be easily implemented and tested while benefiting from shared data preparation and postprocessing routines. Currently, a few software packages are publicly available or available on request [see for instance Favre-Nicolin *et al.* (2011 ▸), Nashed *et al.* (2014 ▸, 2017 ▸), Enders & Thibault (2016 ▸), Marchesini *et al.* (2016 ▸) and Dong *et al.* (2018 ▸)]. With *PtychoShelves*, we provide a toolbox which is geared towards a flexible framework by providing modules for various detector file formats and geometries, as well as different reconstruction algorithms called engines. Similar to a bookshelf, where single books can be read and ordered arbitrarily, *PtychoShelves* does not impose constraints on the order or usage of the reconstruction modules. Beyond its convenience for testing and prototyping new algorithms, *PtychoShelves* also includes high-performance engines for achieving fast ptychographic reconstructions. It therefore enables a fast reconstruction, comparable with or even faster than other currently existing toolboxes, whilst providing a high-level modular framework.

After a description of the general concept of the *PtychoShelves* toolkit, the data types used, and the inputs and outputs in Section 2, the principles of the modular framework are presented in Sections 3 and 4. In Sections 5 and 6 the implementation details of our high-performance CPU and GPU engines are elaborated. Finally, Section 7 provides an overview of *PtychoShelves*’ performance compared with other published toolboxes.

## 2. Processing pipeline   

**Figure 2:**
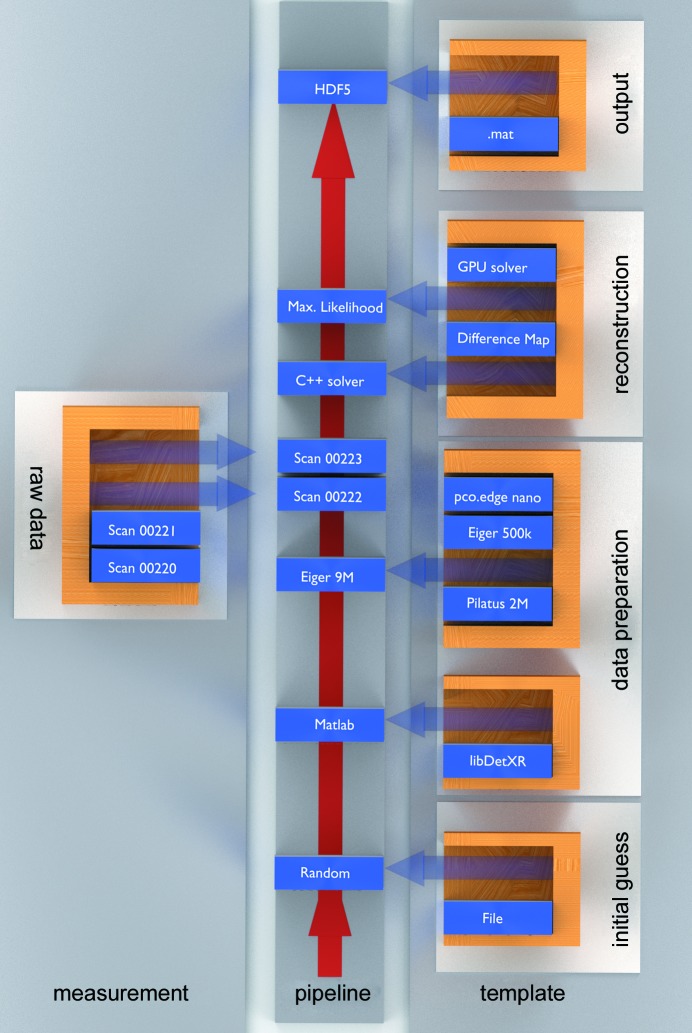
The *PtychoShelves* processing pipeline. Based on the settings of the user-defined template, an initial guess for the reconstructed object and probe can be selected. For subsequent data preparation, the data preparator and detector settings are loaded. After triggering the preparation and loading the measured data, the output is handed over to the engines, where the actual reconstruction is performed. Finally, the result is saved using the specified data format.

*PtychoShelves* is a MATLAB-based (The MathWorks, 2015 ▸) software package designed to take care of the full data pipeline, starting from receiving frames from the detector, through preprocessing and storing intermediate files, to applying iterative algorithms and providing the reconstruction for postprocessing, *e.g.* for tomographic reconstruction. A flowchart can be seen in Fig. 2 ▸.

The data are processed according to a user-defined template, where parameters such as the data preparator, detector settings, reconstruction engines and output file format are specified in the form of MATLAB scripts. This template creates a MATLAB structure, called the *p* structure, which is provided to all *PtychoShelves* modules. Consequently, changes to the *p* structure, and thus to the behaviour of the reconstruction, can be achieved by modifying the settings in any subfunction of the processing pipeline.

### 2.1. Data preparation   

Subsequent to defining all paths and parsing the settings for the data preparator in the initialization process, routines for preparing the data are executed. This includes reading the scan positions, and adjusting the measured diffraction patterns such that the final data set is rotated, shifted and cropped if necessary. Additionally, a valid-pixel mask for the corresponding detector is applied and the probe and object are initialized. The detector data can either be read from a file [HDF5 (The HDF Group, 2018 ▸), CBF (Bernstein & Hammersley, 2006 ▸) or TIFF] or streamed directly via ZeroMQ (Hintjens, 2012 ▸). Alternatively, a virtual detector can be used to simulate measurements. A detailed explanation of the data preparation will be given in Section 3.

The relative positions between sample and probe, as defined in equation (1), can either be parsed from a *SPEC* (Certified Scientific Software, 2017 ▸), ASCII, HDF5 or MATLAB data file, or created according to a user-defined scan pattern in the template.

*PtychoShelves* supports multiple scans to be reconstructed simultaneously, while sharing information about the object or probe (Guizar-Sicairos *et al.*, 2014 ▸). For this purpose two vectors are implemented, probe_share_ID and object_share_ID. These can be seen as vectors of pointers to a slice of the probe and object data sets, respectively, where the number of elements is equal to the number of scans. A probe_share_ID of 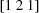 for a simultaneous reconstruction of three scans would therefore result in two probes, one for the first and third scans, and a second probe for the second scan. Similar logic is used for object sharing.

To account for the effects of state mixtures, the concept of probe and object decomposition into modes was introduced (Thibault & Menzel, 2013 ▸). In combination with probe sharing, this results in a 4D array for the probe, (y, x, probe_ID, mode), where y and x represent pixel coordinates, probe_ID indicates the slice of the probe data set and mode the aforementioned illumination mode.

A similar concept was applied to the object storage. Since the reconstructed objects are bound to the extent of the scan range, which can vary for each scan, the object storage was designed as a container of 4D arrays, {object_ID}(y, x, mode, layer), which supports different array sizes. For reconstructions with an extended depth of field where the object is split into multiple planes along the beam direction (Maiden *et al.*, 2012 ▸; Suzuki *et al.*, 2014 ▸; Tsai *et al.*, 2016 ▸), the last index indicates the object layer.

### 2.2. Reconstruction   

After the objects and probes have been initialized and the data preparation has finished, the data are handed over to one of the reconstruction engines. The number of engines used for a reconstruction and their order can be chosen arbitrarily. Additionally, each engine can support independent parameters. The modular framework of the engines’ implementation will be discussed further in Section 4.

### 2.3. Output   

The final reconstruction is stored in the requested data format, either a MATLAB or a general HDF5 file. Specific HDF reading and writing functions were written to achieve a less restricted user experience. *PtychoShelves*’ HDF5 function uses MATLAB’s low-level HDF routines to emulate MATLAB’s proprietary saving routine while being able to write to a user-defined HDF hierarchy and support hard links and symbolic links for internal and external referencing.

**Figure 3:**
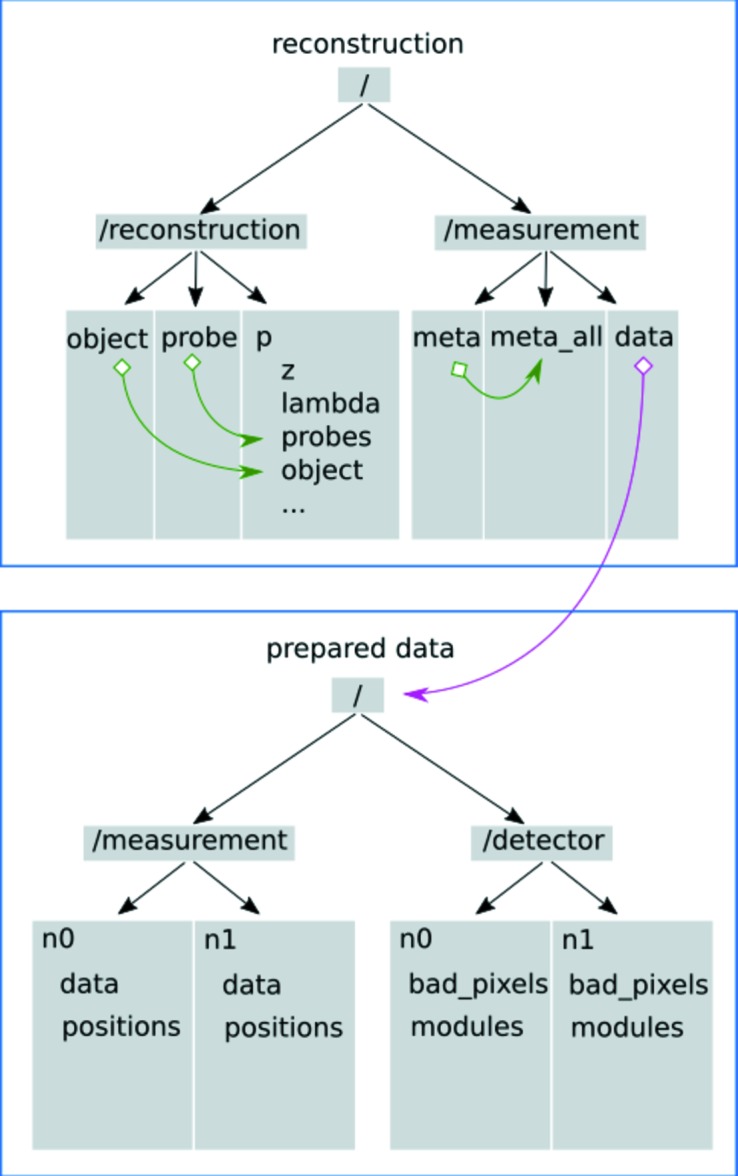
The HDF tree structure for a prepared data file and a single reconstruction file. The prepared data file, written by the data preparator, appends a new group of data and positions for each scan and the corresponding detector settings to the detector group. The reconstruction file uses an external link to provide direct access to the used prepared data, and internal soft symbolic links to access object and probe slices of subscans, indicated by pink and green arrows, respectively.

For each reconstructed scan, a reconstruction file is created. To avoid redundancies, the prepared data are only added as an external link. Furthermore, the reconstruction file uses internal links to provide direct access to the object and probe arrays of the full data set of the corresponding scan, saved in /reconstruction/p/probes and /reconstruction/p/object, respectively. A similar procedure is applied for the metadata of the measurement. In Fig. 3 ▸ the typical structures of prepared data and reconstruction files are shown. This concept allows fast access for further processing.

## 3. Modular data preparation   

To provide a framework which can be adapted quickly to various changes in data sources and formats, as well as to support customized data-processing routines, the preprocessing procedures and data-preparation routines were split into single modules. These modules are grouped into four topics: reading scan positions, reading metadata, supporting different detectors, and creating queues for the reconstruction to share the workload across computation nodes and clusters.

As the functions for reading scan positions and metadata and for creating file queues are independent of each other, they were implemented by single functions in MATLAB packages, a functionality similar to namespaces in other programming languages. For instance, adding the support of a new data structure for reading the scan positions can be achieved by creating an additional function in the +positions package. Due to the differences in reading, preprocessing and data preparation for each detector and file type, the code was split into small subfunctions. Function overriding is employed to cover various scenarios of data preparation.

For each detector, a parameter file is placed in a MATLAB package directory, *e.g.* eiger.m in +eiger, where the details of the detector implementation are specified, including the pixel size, data-loading parameters and readout geometry. For specific modifications related to a single detector, default routines can be overridden by a function in the detector’s package directory without affecting other detector implementations.

For commissioning and testing new detectors in particular, the support of function overriding gives the flexibility to provide fast adaptation without breaking the stability and performance of previous implementations, thus reducing the maintenance to a minimum. In order to reduce the time needed to prepare the data, two routines were implemented to load the data: a modular MATLAB data preparator, based on a multi-threaded C++/MEX function, and *libDetXR* (Zamofing, 2013 ▸), a Python processing toolbox.

## 4. Reconstruction modules   

To provide a consistent environment for further development of methods, each ptychographic reconstruction module receives the same input parameters, including user-defined settings for the current algorithm, specified in the template, and a MATLAB structure containing information about the reconstruction. Changes to the reconstructed object, probe and scan positions are forwarded to the subsequent engine. *PtychoShelves*’ modular framework does not rely on an object-oriented implementation. New engines can easily be added as a single function to the +engines directory. The engine will be provided with already prepared data and initialization parameters, as well as access to all core functionalities, including *e.g.* plotting and saving routines. This concept provides a versatile base upon which various modifications and extensions can be built.

## 5. High-performance CPU engine   

The ease and simplicity of high-level programming languages often comes at the cost of decreased performance. Even with just-in-time compilation (The MathWorks, 2015 ▸), compared with low-level programming languages like C/C++ the performance is often sacrificed for readability and ease of use. However, it is crucial to match the data-acquisition speed and reconstruction time to avoid piling up unprocessed data. In particular when working with brilliant light sources, the acquisition time of a scan can be as short as a few seconds (Celestre *et al.*, 2017 ▸; Klug *et al.*, 2018 ▸; Odstrcil, Lebugle *et al.*, 2019 ▸). It is therefore necessary to combine the aforementioned capabilities with high-performance computing. In *Ptycho­Shelves* this was achieved by implementing the difference map (Thibault *et al.*, 2008 ▸) and a maximum-likelihood refinement (Thibault & Guizar-Sicairos, 2012 ▸) in a standalone C++ binary, using OpenMP and MPI to distribute the workload across CPU cores and distributed memory nodes of analysis clusters (OpenMP Architecture Review Board, 2011 ▸; Message Passing Interface Forum, 2012 ▸). The improved performance of the high-performance CPU engine (HPC) enables online feedback to the user and thus, combined with fast tomography codes (Gürsoy *et al.*, 2014 ▸; van Aarle *et al.*, 2016 ▸; Odstrčil, Holler *et al.*, 2019 ▸), also online feedback on partially measured tomography data sets. HPC has already been exploited in recent publications (Holler *et al.*, 2014 ▸, 2017 ▸; Donnelly *et al.*, 2015 ▸, 2017 ▸; Guizar-Sicairos *et al.*, 2015 ▸; Nielsen *et al.*, 2016 ▸; Wilts *et al.*, 2017 ▸; Ihli *et al.*, 2017 ▸, 2018 ▸; Wakonig *et al.*, 2019 ▸).

### 5.1. Optimizations employed   

To achieve high performance, the C++ code had to be adapted to contemporary hardware. The hardware for which the code was optimized consists of Infiniband-connected computer cluster nodes with two multicore Intel CPUs each. Peak performance can only be reached by utilizing the vector units of each CPU core to the maximum extent. Beyond a sheer optimization of parallel activities, the limited memory bandwidth had to be addressed by adapting the code to the caching mechanisms, hardware prefetching units and memory buses of the system. Additionally, unnecessarily idling vector units might reduce parallelism, which can be caused by both excessive synchronization between small parallel activities and extended wait times between large ones.

The central tenet of the implementation lies in the optimization of the most common scenarios of ptychographic reconstruction on the cSAXS beamline. For this task, the following guidelines have been established:

(i) Parallelization is done by assigning diffraction patterns and associated computations to cores in a fixed way to help caching.

(ii) Cores can try to assist other slower cores in order to reduce idling. Help is first given to cores that are on the same CPU socket.

(iii) Data touched by a core are put into memory close to the core to better utilize the memory buses.

(iv) If possible, computations are split into thread-local computations and a final aggregation step involving synchronization to help parallelization.

(v) Computational tasks on the same data are fused into bigger code sections to assist caching.

(vi) Computations are done on contiguous memory blocks to help memory prefetching.

(vii) Memory blocks are aligned and sized to fit vector sizes and cache lines in order to help memory handling.

(viii) Computations are vectorized explicitly where this proves to be faster than compiler-generated code.

(ix) Distributed memory nodes all keep a copy of the probes, but the object memory is distributed.

### 5.2. Program options, input and output   

As input, the HPC binary receives a description of the measurement-related data such as diffraction patterns and positions, and a description of the initial approximation for objects and probes which the code is supposed to improve. Data transfer between MATLAB and HPC can be achieved via either a TCP/IP stream or two HDF5 files. For the latter, the decision to have two files was made to reduce the data processing if only reconstruction parameters change. Therefore, the prepared data and the initial guess are written in separate files. The main output of all code variants is either an HDF5 solution file or a TCP/IP stream with updated object and probe arrays, which represent the object’s transmissivity and the illumination, respectively.

### 5.3. Difference-map and maximum-likelihood implementation   

We have adopted the difference-map (DM) method described by Thibault *et al.* (2008 ▸). By performing the Fourier projection first, followed by the overlap projection, the difference-map implementation reverses the order of the projections compared with the approach used by Thibault *et al.* (2008 ▸). Assuming a good initial guess, this reduces the reconstruction time by essentially skipping the first overlap projection. The error metric used for DM is the sum of the differences between the current model’s amplitude at the detector plane [equation (2)] and the measurement, calculated for all valid pixels *l* ∈ *M* and positions *k* ∈ *N*,
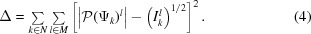



The second method, a maximum-likelihood refinement (Thibault & Guizar-Sicairos, 2012 ▸), uses a conjugated gradient optimization. The search directions are calculated using the Pollak–Ribière *Plus* method with a Powell restart criterion (Andrei, 2010 ▸; Hager & Zhang, 2006 ▸). The line search first attempts to establish a bracket around the solution. New solution points within the bracket are probed using quadratic and cubic fits. If these methods fail, a geometric bisection search for better solution points within the bracket is tried. The Armijo (1966 ▸) criterion is used to decide whether a new solution point is good enough. However, due to the loss of significance in floating-point summation, an accurate calculation can be numerically challenging. Therefore, a special summation algorithm was implemented. By grouping and summing values with approximately the same magnitude, the adverse effect of limited precision can be mitigated.

### 5.4. OpenMP implementation   

The main decision for the OpenMP code is the fixed assignment of diffraction patterns and corresponding views to OpenMP threads. The code assumes one pinned thread per core, which can be achieved by setting the OMP_PROC_BIND environment variable (OpenMP Architecture Review Board, 2011 ▸). As a result, multiple calculations can be performed in parallel on data closely located in memory to the corresponding cores. However, due to the intrinsic parallelization difficulties of ptychography, as the same memory location has to be accessed by multiple instances, the threads need to coordinate actions. This affects the probe and object update phases of the overlap projection in the difference-map reconstruction algorithm, as well as the error metric value aggregation, search direction calculation and gradient calculation steps of the maximum-likelihood reconstruction algorithm. Moreover, probe and object updates, as well as the gradient calculation, rely on synchronization-heavy operations, where contributions in the form of submatrices for all threads need to be summed into a process-wide matrix.

**Figure 4:**
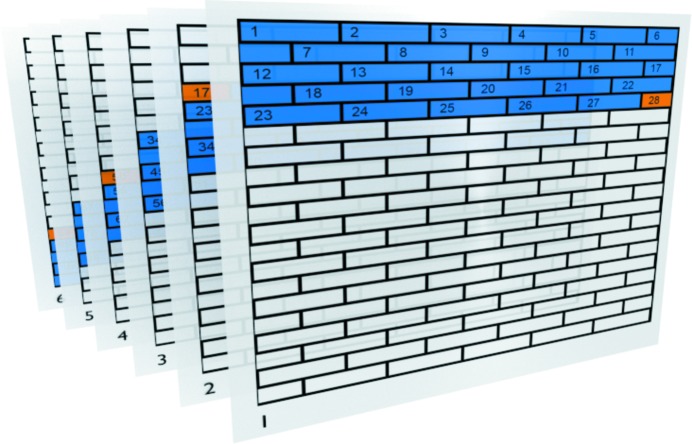
The OpenMP reduction algorithm scheme for six threads. The object array is subdivided into submatrices (blue) and distributed between the threads, shown as object layers 1–6. Each submatrix is again subdivided into smaller blocks of 64 cache lines and a consecutive sequence number (*e.g.* 1–28 for the first thread). On each addition, a thread fetches the current sequence counter and increases it atomically. To avoid having multiple threads working on the same array block, a reservation lock is used. If blocks are not fully covered (orange), they are treated separately.

In order to reduce idling of cores at OpenMP synchronization barriers and perform as many operations as possible in memory close to the core, a special reduction algorithm has been devised (Fig. 4 ▸). The process-wide matrix is split up into a sequence of consecutive aligned data blocks, in our case comprising 64 cache lines. For each of these blocks, there is a reservation lock that needs to be held exclusively by a thread when working on that particular data block. The submatrix of each thread consists of a range of entire rows of the process-wide matrix. To minimize the overlap of the submatrices, views are initially sorted by row position within the object for the purpose of assigning views and the corresponding diffraction patterns to threads.

A submatrix is split into blocks, namely the blocks that the corresponding row range covers. If the first and last blocks are not fully covered, they are treated separately. Each thread has an atomic counter, initialized to the sequence number of the first fully covered block in the submatrix.

On each addition of a submatrix block onto the corresponding process-wide matrix block, every thread fetches the counter value and increases it atomically. The fetched counter value is the sequence number of the submatrix block that can be handled exclusively by the thread after obtaining the reservation lock for the corresponding block in the process-wide matrix. If a thread has finished with its submatrix, that thread can be employed to assist in processing the submatrices of slower threads. In order to stay close to the handled memory, slower threads residing on cores in the same CPU are preferred in this process.

### 5.5. OpenMP/MPI hybrid implementation   

Following the same principles as for the OpenMP implementation, diffraction patterns and the corresponding views are assigned to MPI processes to perform calculations in parallel on data located in a node’s local memory. This fixed assignment provides a way of distributing the object matrix data between the nodes. Each node only keeps the range of object matrix rows covered by the views assigned to the MPI process. The decision to keep a range of entire rows, rather than a smaller submatrix, was made in order to reduce the complexity of the MPI communication and synchronization patterns. To sum the contributions of all MPI processes covering a stripe of rows, the MPI_Allreduce operation is performed on the stripe among all the MPI processes covering the stripe (Message Passing Interface Forum, 2012 ▸).

### 5.6. HPC engine performance evaluation   

For performance measurements, the code was run on a computing cluster at the Paul Scherrer Institute, Switzerland. The nodes used have two sockets with 16 cores each (hyperthreading disabled) equipped with Intel Xeon E5-2697A v4 CPUs running at 2.60 GHz and a maximum bandwidth of 76.8 GB s^−1^ per socket. Cluster nodes are connected via Mellanox ConnextX 3 FDR Infiniband and run CentOS 7.4 as the operating system. The code was compiled with gcc 7.3.0, openmpi 3.0.0 and Intel MKL 2018.0.0. Performance data were sampled with a frequency of 3.3 Hz using the *likwid-perf* tool (Version 4.3.0; Treibig *et al.*, 2010 ▸; Roehl *et al.*, 2014 ▸). To show the scalability properties of the OpenMP implementation, we used a data set with 423 positions, a probe of size 512 × 512 pixels and an object of size 2649 × 2644 pixels. Two illumination modes were reconstructed. We performed 300 difference-map iterations followed by a maximum-likelihood refinement. For the difference-map algorithm, the time spent for each iteration is comparable. However, iterations within the maximum-likelihood refinement may vary considerably due to the line search step in the high-dimensional solution space. We therefore used the maximum-likelihood refinement until a specific error value was reached, resulting in a comparable reconstruction between single runs.

**Figure 5:**
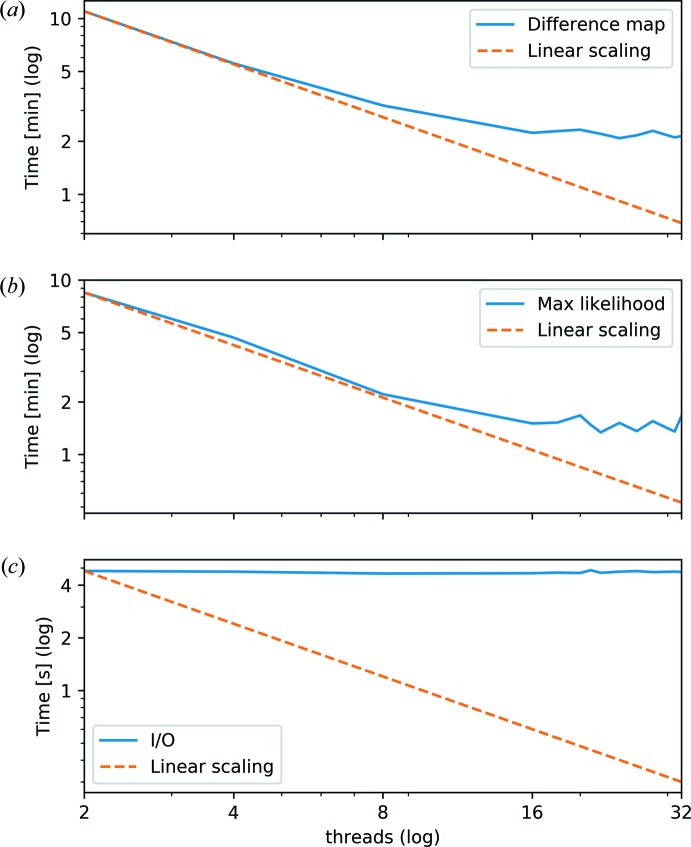
(*a*) The time to complete 300 difference-map iterations for different thread counts on a single node. (*b*) The time for the maximum-likelihood refinement to reach a specific error value limit for various thread counts on a single node. (*c*) The I/O time to read the data and write the reconstruction result for various thread counts. Due to the use of a single-threaded HDF5 library, no performance gain was achieved by using more than one thread during I/O. For (*a*) and (*b*), the performance benefits of more threads are clearly visible. The increased synchronization time in the case of many threads, as well as the limited memory bandwidth, limits the performance gain in the case of more than 20 threads.

The results shown in Fig. 5 ▸ highlight the scalability limitations. One of the limits that cannot be overcome by using more threads on the same computer node is the memory bandwidth. Moreover, the synchronization time increases with more threads. Our implementation suffers from an additional limitation due to the aggregation mechanism based on stripes of rows. Assuming a more or less equal distribution of measurement positions, the minimum number of views to form a reasonably covered stripe of rows is around 20 for the used data set. Therefore, the usage of more than 21 threads leads to stripes of rows which are not fully covered by the views. The stripe of a particular thread will thus contain regions of zero values, which are nevertheless aggregated into global matrices, essentially wasting memory bandwidth. In the future, this could be addressed by changing the per-stripe atomic block reservation counter in the aggregation method to a per-row counter, only swiping through covered blocks.

For larger data sets, additional performance can be gained by using the aforementioned OpenMP/MPI hybrid implementation. The reconstructed data set had 11 844 positions, 28 probes of size 500 × 500 pixels and one object of size 8058 × 13 525 pixels, resulting in an uncompressed total size of 11.9 GB (Guizar-Sicairos *et al.*, 2014 ▸). For each probe two illumination modes were reconstructed. The data set can be seen as a combination of 28 separate data sets to achieve an extended field of view. Although such large data sets are not very common on the cSAXS beamline, the sheer quantity of data highlights bottlenecks in the data-processing routines.

**Figure 6:**
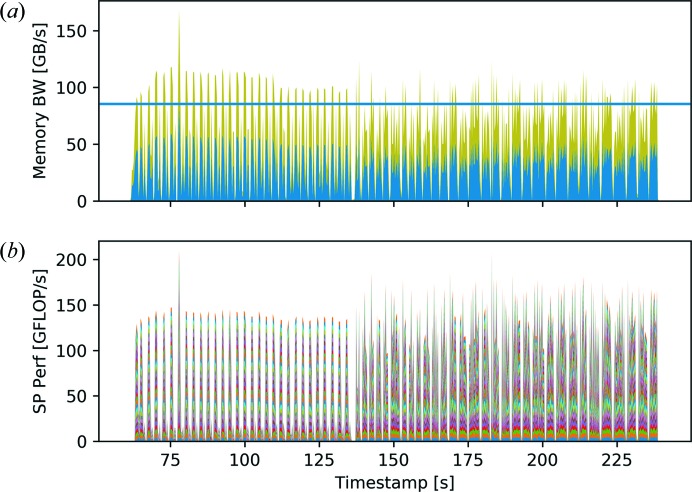
(*a*) Aggregated memory bandwidth and (*b*) single-precision computation performance for the first node of a hybrid run using ten cluster nodes with 32 threads each. The stream benchmark memory bandwidth result for 32 threads has been added as a reference (horizontal blue line). Different colours are used for each thread. Only one thread per socket reports on the memory bandwidth since these data are based on uncore events.

For the first test, ten nodes with 32 threads each were allocated. After the data had been read and initialized, 30 difference-map iterations followed by 30 maximum-likelihood iterations were performed before the result was written to the file system. Fig. 6 ▸ shows the measured memory bandwidth and computer performance data for the first node. Additionally, the stream benchmark for 32 threads measured with the *likwid-bench* *stream-sp-avx* benchmark code is given as a reference (Treibig *et al.*, 2012 ▸). Using one work group, the stream bandwidth was 87.64 GB s^−1^. This benchmark reflects the data-access patterns of the various computational loops in the code rather well. A vector addition benchmark with operations exclusively on aligned thread-local data achieved a bandwidth of 122.38 GB s^−1^ with 32 threads. The theoretical maximum computational performance 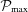 of a cluster node is given by
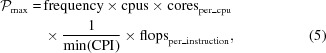
where CPI denotes the clock count per instruction. With a frequency of 2.6 GHz, two sockets, 16 cores per CPU, eight single-precision floating-point operations per AVX multiply instruction and a minimal clock count per AVX multiply instruction (CPI) of 0.5,^2^ this amounts to approximately 1.33 Tflop s^−1^ for multiplications. If computations could be carried out with independent fused multiply–add instructions (CPI 0.5 and 16 flops per instruction) exclusively, this number would double. For addition this number would halve, since the minimum CPI is 1 in this case for the Broadwell architecture (Intel Corporation, 2018 ▸).

It must be noted that floating-point operations often depend on previous computations and may be further limited by slow memory load operations. Both the difference-map and the maximum-likelihood reconstruction methods suffer from these problems. The typical loop gathers elements from a few matrices and computes one or two values that are written into a matrix or accumulated. As illustrated in Fig. 6 ▸, the computation performance graph shows peaks well below the theoretical peak computation performance. At the same time, the memory bandwidth graph shows peaks that are around or even above the stream benchmark results. Since the computations are simple element-wise operations, the memory bandwidth is the main bottleneck for both reconstruction methods. However, the OpenMP thread and distributed MPI process synchronization time impose additional constraints on the achievable performance, visible as white gaps between peaks in both graphs of Fig. 6 ▸.

**Figure 7:**
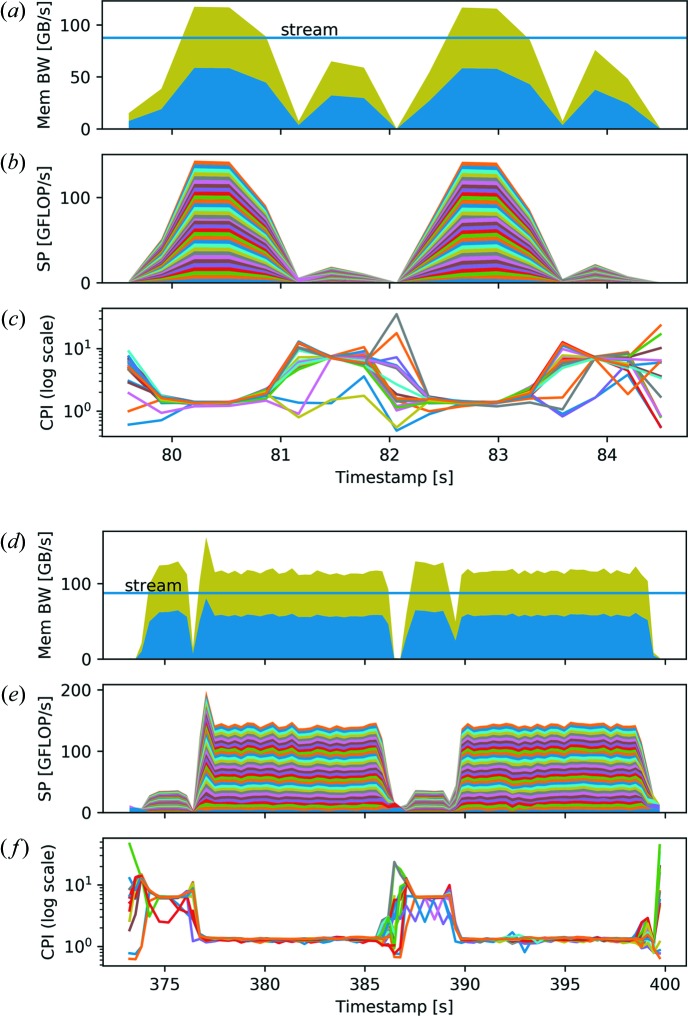
(*a*)–(*c*) Enlargements of the difference-map interactions of Fig. 6 ▸, running on ten nodes with 32 threads each. (*d*)–(*f*) Enlargements of the difference-map iterations on a single node with 32 threads. For each run, (*a*), (*d*) the memory bandwidth, (*b*), (*e*) the single-precision performance and (*c*), (*f*) the clocks per instruction (CPI) are shown. The CPI can be seen as efficiency measurements and are given for each thread of the first node. The increase in synchronization time in the case of multiple nodes and the resulting rise in CPI is clearly visible. Furthermore, the Fourier projection shows a significantly improved performance compared with the overlap projection, *e.g.* from timestamp 80 s to 81 s in panels (*a*)–(*c*) and from 81 s to 82 s in panels (*a*)–(*c*), respectively. These effects can also be attributed to the increased synchronization time between threads.

In general, synchronization patterns are a result of the combination of algorithm and parallelization. For the difference-map method and the chosen parallelization with a fixed distribution of diffraction patterns to threads, synchronization is required to maintain a common view of the objects and probes in the overlap projection. Similarly, synchronization is required if feedback on the conversion is needed. However, the possibilities for hiding the synchronization overhead without introducing extra synchronization, *e.g.* by performing independent computations while MPI communication is done in the background, are limited. The performance loss cannot be addressed by allocating additional resources, as more threads and computer nodes will increase the synchronization time further. It therefore imposes a fundamental limit on the scalability. Fig. 7 ▸ shows enlargements of the difference-map iterations on ten nodes [Figs. 7 ▸(*a*)–7 ▸(*c*)] and one node [Figs. 7 ▸(*d*)–7 ▸(*f*)]. For both runs, the achieved CPI for every thread is shown. The CPI numbers in the computationally heavy loops are well above 1, indicating that the computational resources are not fully used. Moreover, as an effect of using more nodes, the increased computation power results in narrower peaks. At the same time, the white spaces due to synchronization make up a larger part of the graphs in Figs. 7 ▸(*a*) and 7 ▸(*b*) compared with 7 ▸(*d*) and 7 ▸(*e*).

**Figure 8:**
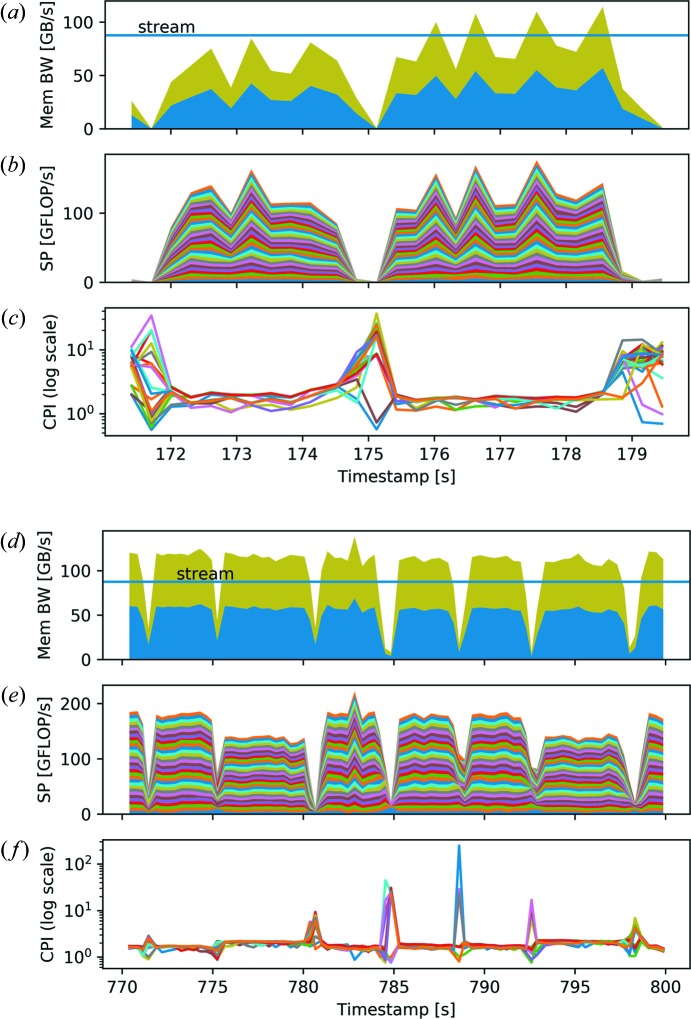
(*a*)–(*c*) Enlargements of the maximum-likelihood iterations of Fig. 6 ▸, running on ten nodes with 32 threads each. Additionally, the efficiency measure CPI is given for every thread. (*d*)–(*f*) Enlargements of the maximum-likelihood iterations on a single node run with 32 threads. Similar to Fig. 7 ▸, (*a*), (*d*) the memory bandwidth, (*b*), (*e*) the single-precision performance and (*c*), (*f*) the CPI are shown for each run.

The same observations hold for Fig. 8 ▸, which shows subsections of the maximum-likelihood refinement for runs with ten nodes and one node, 32 threads each. For the maximum-likelihood method using the conjugated gradient algorithm and the chosen parallelization with a fixed distribution of diffraction patterns to threads, synchronization is required to maintain the common view of the gradient, to coordinate the line search, and to compute the error function value and a number of dot products for the search direction update.

To show absolute reconstruction time measurements and scaling properties, the reconstruction problem was first handled with 300 difference-map iterations, followed by maximum-likelihood refinement iterations until a specific error value limit was reached. This value was used to ensure that the reconstruction quality could be considered similar to that of previous reconstructions.

**Figure 9:**
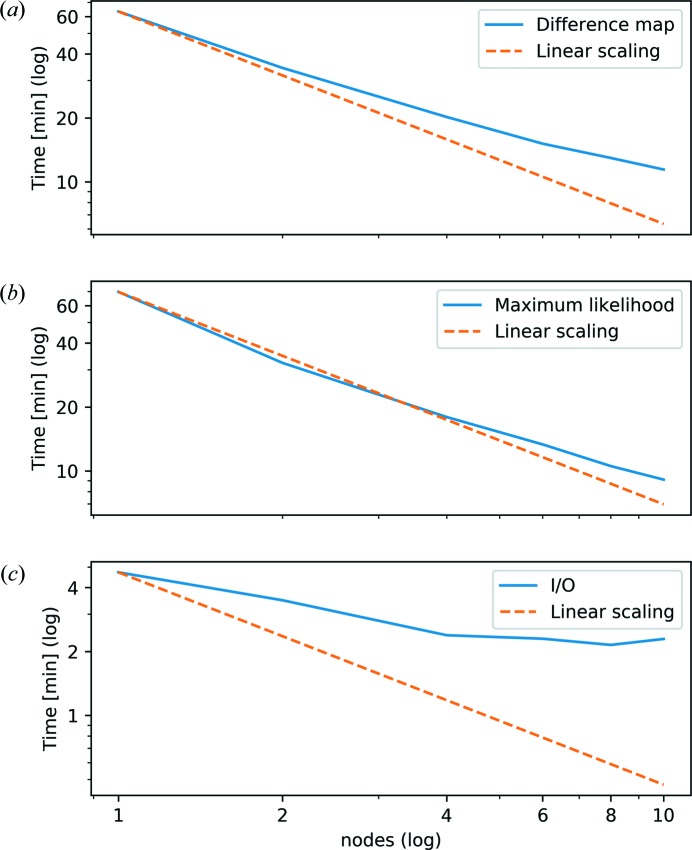
(*a*) The time to complete 300 difference-map iterations for various node counts and 32 threads per node. (*b*) The time for the maximum-likelihood refinement to reach a specific error value limit for various node counts and 32 threads per node. (*c*) The I/O time to read the data and write the reconstruction result for various node counts and 32 threads per node. The effect of increased synchronization time for larger numbers of nodes is clearly visible.

Fig. 9 ▸(*a*) gives the time to complete 300 iterations of the difference map. The time assuming perfect linear scaling is added as a comparison. Difference-map iterations are quite comparable across runs with different node counts, except for slight numerical differences and jitter due to the number of nodes and thread scheduling, as well as general OS jitter. However, an increase in synchronization time clearly leads to reduced efficiency for a higher number of nodes. Iterations for the maximum-likelihood refinement are less comparable across runs and node counts, as even minor numerical differences can multiply considerably when walking through the high-dimensional solution space. Therefore, the number of iterations to reach the specific error value limit varied from 173 to 217 for the presented runs. The reconstruction time for the maximum-likelihood refinement is shown in Fig. 9 ▸(*b*).

As expected, the part that scales the least is the part that handles data input and output (I/O) [Fig. 9 ▸(*c*)]. The measurement data file and the initial solution approximation were read as HDF5 files from a GPFS file system. The reconstruction result was written to the same GPFS file system, also in the form of an HDF5 file. Nonetheless, the total time spent on I/O was only a small fraction of the total reconstruction time.

## 6. MATLAB-based GPU-accelerated engine   

Although usually running at a lower clock frequency than a CPU, the sheer number of cores on a GPU device allows for significantly improved performance if calculations can be run in parallel. *PtychoShelves* exploits these advantages with a MATLAB-based GPU-accelerated (MG) engine, which provides a performance close to, or even better than, the highly optimized CPU code presented in Section 5. The implementation relies mostly on high-level built-in MATLAB functions and comprises a collection of various reconstruction algorithms and refinement methods which can be easily combined and chained: difference map (DM) (Thibault *et al.*, 2008 ▸), extended ptychographic iterative engine (ePIE) solver (Maiden & Rodenburg, 2009 ▸) and iterative least-squares maximum-likelihood (LSQ-ML) (Odstrčil *et al.*, 2018 ▸). In particular, this last provides many additional refinement directions such as position and geometry refinement, variable wavefront and intensity refinement, and multilayer ptychographic reconstruction for extended depth of field (Odstrčil *et al.*, 2016 ▸, 2018 ▸; Tsai *et al.*, 2016 ▸). Additionally, mixed-state modes (Thibault & Menzel, 2013 ▸) and a method of accounting for incoherent background signal (Odstrčil *et al.*, 2015 ▸, 2018 ▸) are implemented for all MG engine methods. The combination of fast GPU-based methods with high-level scripting was also used to facilitate the implementation of a broad range of wavefront propagation methods. In addition to the conventional far-field and Fresnel propagator, the MG engine supports the angular spectrum method (ASM) for near-field propagation, the fractional Fourier transformation (FFTR) (Ozaktas *et al.*, 1996 ▸), fast Fourier transform (FFT)-based rotation (Larkin *et al.*, 1997 ▸) and propagation to a tilted plane (Delen & Hooker, 1998 ▸). Furthermore, these propagators can be combined to perform *e.g.* a propagation to a tilted and rotated plane placed in the near-field propagation regime. If no suitable GPU hardware is available, the MG engine automatically falls back to a CPU implementation.

### 6.1. Implementation   

For the most common numerical operations used in ptychography, such as wavefront propagators and element-wise operations, MATLAB built-in GPU-accelerated functions can be used. To further accelerate the reconstruction, multiple views can be grouped into blocks and processed in parallel. Their overall size, however, needs to be small enough to fit into the available GPU memory and sufficiently large to avoid additional overhead caused by the launch of the CUDA (https://developer.nvidia.com/cuda-zone) kernel before each GPU operation.

The MG engine automatically estimates the memory requirements for each of the implemented methods with its extensions and chooses the optimal parallel block size. This provides high computational efficiency for a broad range of ptychographic experiments. In contrast to the HPC engine, which supports MPI and thus communication across multiple nodes, the GPU engine is currently limited to a single device. Therefore, the reconstruction time and the maximum size of a reconstruction are determined by the computational power and memory of a single GPU.

To optimize the performance further, the communication, that is the data transfer from CPU to GPU memory, has to be minimized. Therefore, the engine was designed such that the only required large-volume CPU–GPU communication is the initial upload of the measured data into the GPU memory.

### 6.2. Data compression for ptychography   

**Figure 10:**
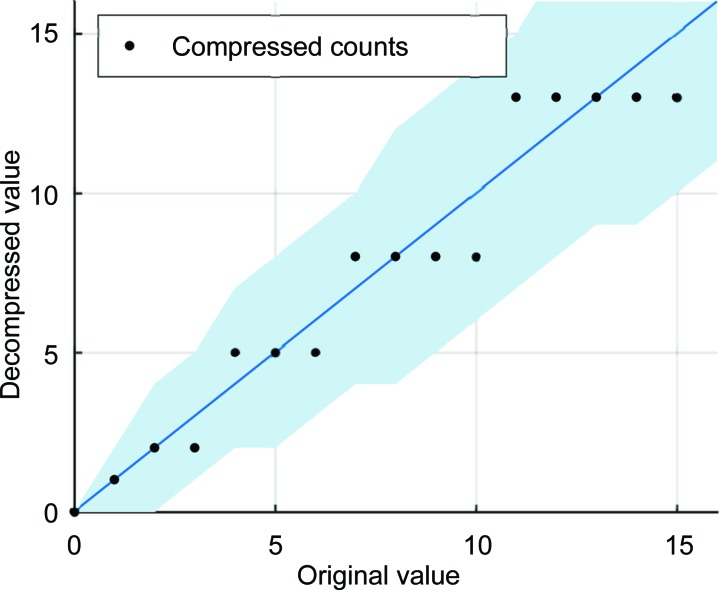
Examples of decompressed values versus the original count number for quantization step QS = 0.5. The blue shaded area denotes the range containing 90% of the Poisson-distributed values. Black dots denote the dependence of the decompressed counts on their original value *N*_*i*_.

By keeping the measured data in GPU memory, the maximum size of the data sets can become a limitation of the reconstruction dimensions. To lessen these constraints, an online lossy compression of the measured data was implemented. The compression method is based on the assumption that the measured diffraction patterns are well described by Poisson statistics. The measurements were rescaled by a simple variance stabilization transformation,
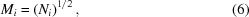
where *N*_*i*_ denotes the number of captured photons or electrons. For ideal Poisson-noise-limited data sets, the standard deviation of the transformed measurements *M*_*i*_ can be well approximated by σ = 0.5. If there are additional sources of noise, the value of the standard deviation will only increase. Therefore, the compression itself is performed by optimum quantization of the transformed value *M*_*i*_. If the quantization error of the lossy compression is sufficiently lower than the error caused by the Poisson statistics (Fig. 10 ▸), the *M*_*i*_ values can be stored in a lower-precision format with negligible information loss.

**Figure 11:**
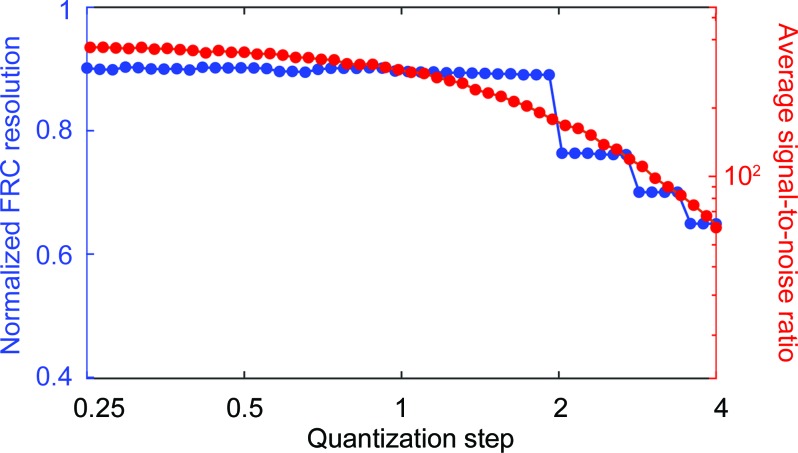
Reconstruction quality for a simulated ptychographic data set limited by Poisson noise, plotted versus the quantization step QS. The reconstruction quality was measured by means of the normalized spatial resolution determined by a Fourier ring correlation (FRC), as well as by the signal-to-noise ratio between the original model and the reconstruction.

The compression scheme uses the following formula to store the measured intensities into 8 bit unsigned integer values 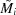:
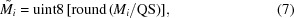
where QS denotes the quantization step. Fig. 10 ▸ highlights the introduced errors compared with Poisson noise for values of *M*_*i*_ from 0 to 16 counts. The dependence of ptychographic reconstruction quality for a simulated Poisson-noise-limited data set is shown in Fig. 11 ▸. Using the lossy compression scheme, measured intensities of up to 16 384 counts can be stored in 8 bit unsigned integer data format. If the measured data exceed 16 384 counts, the 16 bit unsigned integer format will extend the range up to 10^9^ counts per pixel. Yet, as such high intensities are rare, the memory allocation can be reduced by up to 75% of what would otherwise be required to store the modulus values in the commonly used 32 bit single-precision format. Using online compression, 1000 measured diffraction patterns of size 1024 × 1024 occupy a mere 1.04 GB of GPU memory. Therefore, using the LSQ-ML method, a single GPU with 16 GB memory is sufficient to reconstruct a 98.4 megapixel ptychographic data set (Guizar-Sicairos *et al.*, 2014 ▸).

## 7. Overview of engine performance   

There is no ideal way to compare computational performance between various engines, other toolkits based on different hardware, and reconstruction methods which may differ significantly in their convergence speed. However, providing at least a basic comparison is important for understanding the limitations of different reconstruction approaches. For this task, the performance was evaluated by measuring the computation time for three different ptychographic data sets. The first data set contains 253 scan positions with a probe size of 256 × 256 pixels, the second one contains 8998 positions with a probe size of 256 × 256 pixels and the third contains 1569 positions with a probe size of 1024 × 1024 pixels.

Table 1 ▸ shows a comparison of the computation times normalized by the number of iterations, scan positions and pixels of the probe. Using a single computational node, the HPC engine can compete well with the GPU-based implementation for smaller data sets. With larger data sets, the overhead caused by the CUDA launch becomes negligible compared with the computation time and the difference between the engines becomes dominated by computational speed and memory bandwidth only. Additionally, the LSQ-MLc method (Odstrčil *et al.*, 2018 ▸) is computationally less expensive than the ML method (Thibault & Guizar-Sicairos, 2012 ▸), mainly due to the absence of the line search required for conjugate gradient optimization, as discussed in Section 5.3. For this reason, the LSQ-ML method can be up to eight times faster than the HPC ML method. Nevertheless, the HPC engine’s support for distributing the computation across multiple nodes can significantly reduce the required time to finish a single reconstruction, as shown in Fig. 9 ▸. Table 1 ▸ also highlights the fact that the limited GPU memory constrains the maximum reconstructed data-set size for our GPU-based difference-map implementation. Such limitations can be lessened if the DM method is only used as a low-resolution initial guess (Odstrčil *et al.*, 2018 ▸). The memory requirements of the other methods implemented in the MG engine depend only on the size of the parallel blocks and the volume of measured data, which can be significantly reduced by online data compression, as described in Section 6.2.

In order to demonstrate the performance of the *PtychoShelves* engines with respect to other implementations, we provide in Table 2 ▸ the normalized computation times for other published ptychography toolboxes, and also predictions for the Nvidia V100 card. The predictions can be used as a rough estimate of the computational cost per iteration. However, the computational cost may differ for various algorithms, or even for identical methods with different reconstruction parameters. Moreover, each method can lead to significant differences in the convergence speed. Additionally, the computation time does not depend linearly on the probe size, although the nonlinearity is rather weak. For our MG-engine GPU engine, the difference is only 20–30% between probe sizes of 256 × 256 and 1024 × 1024 pixels.

Based on the comparisons in Tables 1 ▸ and 2 ▸, for data sets 1 and 2 both our CPU-based HPC and GPU-based MG engines reach shorter per-iteration computation times than other toolboxes would if they were run on an Nvidia V100 GPU. The exception is the *SHARP* toolbox, which could provide comparable single-GPU performance to our MG engine. Also, all the GPU-based methods generally perform better than the CPU-based HPC engine for data sets with a large probe size, as tested with data set 3.

## 8. Conclusions   

*PtychoShelves* has been successfully tested on, and with data from, various synchrotron beamlines, including beamlines at SLS, MAX IV, SOLEIL and ESRF, and laboratory experiments in the visible and extreme ultraviolet range, as well as for electron ptychography. The presented benchmarks of the *PtychoShelves* C++ solver suggest that the main speed limitation arises from the finite memory bandwidth and synchronization overhead. As a result, we are currently investigating ways of porting the C++ solver to a GPU, from which we expect an additional performance gain.

The already existing GPU implementation in MATLAB provides a fast yet flexible environment for method development. Given that large-scale synchrotron facilities around the world expect to increase their coherent flux by orders of magnitude over the next few years, the demands on ptychographic data processing will increase significantly. By essentially providing both a modular framework and high-performance engines, scalable even for large computing clusters, we expect that *PtychoShelves* will be of particular interest for researchers working on ptychography.

*PtychoShelves* is publicly available (CXS, 2019 ▸). For demonstration purposes, the raw data of the results presented in this article can be downloaded, and the data can be reconstructed using a demonstration MATLAB script. *PtychoShelves* comes with a royalty-free non-exclusive licence for academic and non-commercial purposes only.

## Figures and Tables

**Table 1:** Comparison of computation time (ns) required per iteration, pixel and scan position for three different ptychographic data sets and the different engines and methods implemented in our toolkit The CPU-based engines were tested using 2 × 14 cores of Intel Xeon CPU E5-2690 v4 and 512 GB RAM clocked at 2400 MHz. The GPU calculations were performed on a single Nvidia Tesla V100 GPU accelerator with 16 GB memory using MATLAB 2018a.

Engine	Hardware	Method	Data set 1	Data set 2	Data set 3
HPC	2× Intel Xeon CPU E5-2690	DM	1.30	1.31	2.41
HPC		ML	2.41	2.48	4.90
MG		DM	18.5	17.5	16.7
MG	Nvidia GPU Tesla V100	DM	1.03	0.70	Memory limited
MG		MLc	0.81	0.49	0.60
MG		PIE	0.78	0.39	0.50

**Table 2:** A summary of computational performance for *Ptycholib* (Nashed *et al.*, 2014 ▸), *SHARP* (Marchesini *et al.*, 2016 ▸), *PyNX* (Mandula *et al.*, 2016 ▸), the software package of NSLS-II (Dong *et al.*, 2018 ▸) and *ADP* (Nashed *et al.*, 2017 ▸) We provide the normalized computation time as in Table 1 ▸ and a prediction for Nvidia V100 GPU based on the assumption that computational performance is fully memory bandwidth limited. Note that, if the memory bandwidth difference between Nvidia V100 and the used GPU card is large and there are other limitations such as CUDA kernel launch overheads, the predicted computation time may be over-optimistic.

	*Ptycholib*	*SHARP*	*PyNX*	NSLS-II	*ADP*
Hardware	Nvidia M2070	Nvidia Titan X	Nvidia Titan X	Nvidia P100	Nvidia K80
Memory bandwidth (GB s^−1^)	150	337	337	732	480
Method	ePIE	RAAR (no probe update)	DM	DM	*ADP*
Probe size	256 × 256	128 × 128	336 × 336	200 × 200	128 × 128
Normalized time (ns)	9.3	1.4	2.5	2.5	3.6
For V100 (ns)	1.6	0.5	0.9	2.0	1.9

## Footnotes

2 According to the Intel *Intrinsics Guide* (Intel Corporation, 2018 ▸) the Intel Broadwell architecture can simultaneously complete two independent AVX multiplication and fused multiply–add instructions per clock cycle.
